# Supplementary material for: Shared multicellular injury programs of acute and chronic kidney disease enable mechanistic patient stratification
Source: medRxiv. 2026 Mar 6:2026.03.05.26347522. Preprint. [Version 1] doi: 10.64898/2026.03.05.26347522 (PMC12976899; doi:10.64898/2026.03.05.26347522)
Supplement: Supplement 1 [file media-1.pdf]

Supplementary Methods

MCFA model including healthy reference samples

We apply MCFA on all samples included in the snRNA and scRNA data of the Human Kidney Atlas v2[23], including healthy reference samples. Preprocessing steps, including filtering of samples and cell types, and MCFA model fit are done as described in the main methods.

Projection of external dataset

We use the single cell data from a cohort with healthy reference samples obtained through percutaneous biopsy[101], and project these samples by matrix multiplication into the factor space built by HKA v2 scRNA data. This dataset comprises samples from Impact of Metabolic Surgery on Pancreatic, Renal and Cardiovascular Health in Youth with Type 2 Diabetes (IMPROVE-T2D, NCT03620773), Renal Hemodynamics, Energetics and Insulin Resistance in Youth Onset Type 2 Diabetes (Renal HEIR, NCT03584217) Control of Renal Oxygen Consumption, Mitochondrial Dysfunction, and Insulin Resistance (CROCODILE, NCT04074668) studies. For the projection, we take the pseudo-inverse of the loadings matrix (from KPMP scRNA) and multiply it by the normalized, log1p-transformed and then centered multicellular gene expression matrix of the external dataset to obtain the factor scores for the projected samples. In that process, we manually matched cell types between the

Deriving of ischemia signature

To identify a consensus ischemia signature across omics and cell types, we used a rank-based approach, where gene loadings for each cell type were ranked by value, and then averaged across snRNA/scRNA. Assuming that ischemia affects all cell types similarly, we then averaged the ranks across cell types to obtain a consensus ranking, further selecting genes that were measured consistently across at least 3 cell types. The top 50 genes with the highest average rank were selected as ischemia signature genes.

To show that factor scores yield a similar signature to the one from Menon et al.[28], we show that their gene signature is enriched in the factor loadings from snRNA using ORA, and that the genes from their signature are highly ranked in our consensus ranking using GSEA's running score, both using the decoupler-py package[92].

Ischemia signature in bulk datasets

We selected previously published bulk RNA and proteome studies that use control samples obtained through nephrectomy[102–107] and that report differentially expressed gene probes, genes and/or differentially regulated proteins. Given that most studies only report differentially expressed probes/genes/proteins, we used Fisher's exact test – implemented in scipy[108] – to assess the enrichment of our ischemia signature in their reported downregulated probes/genes/proteins. The studies used different analytical approaches and thresholds to define differential expression, and we used the reported p-values and thresholds from each study without re-analyzing the data. When available, we use the reported feature space used by the study as background for Fisher's exact test, otherwise we use the feature space size reported by the study or measured by the platform (e.g., microarray).

Supplementary Figures and Tables

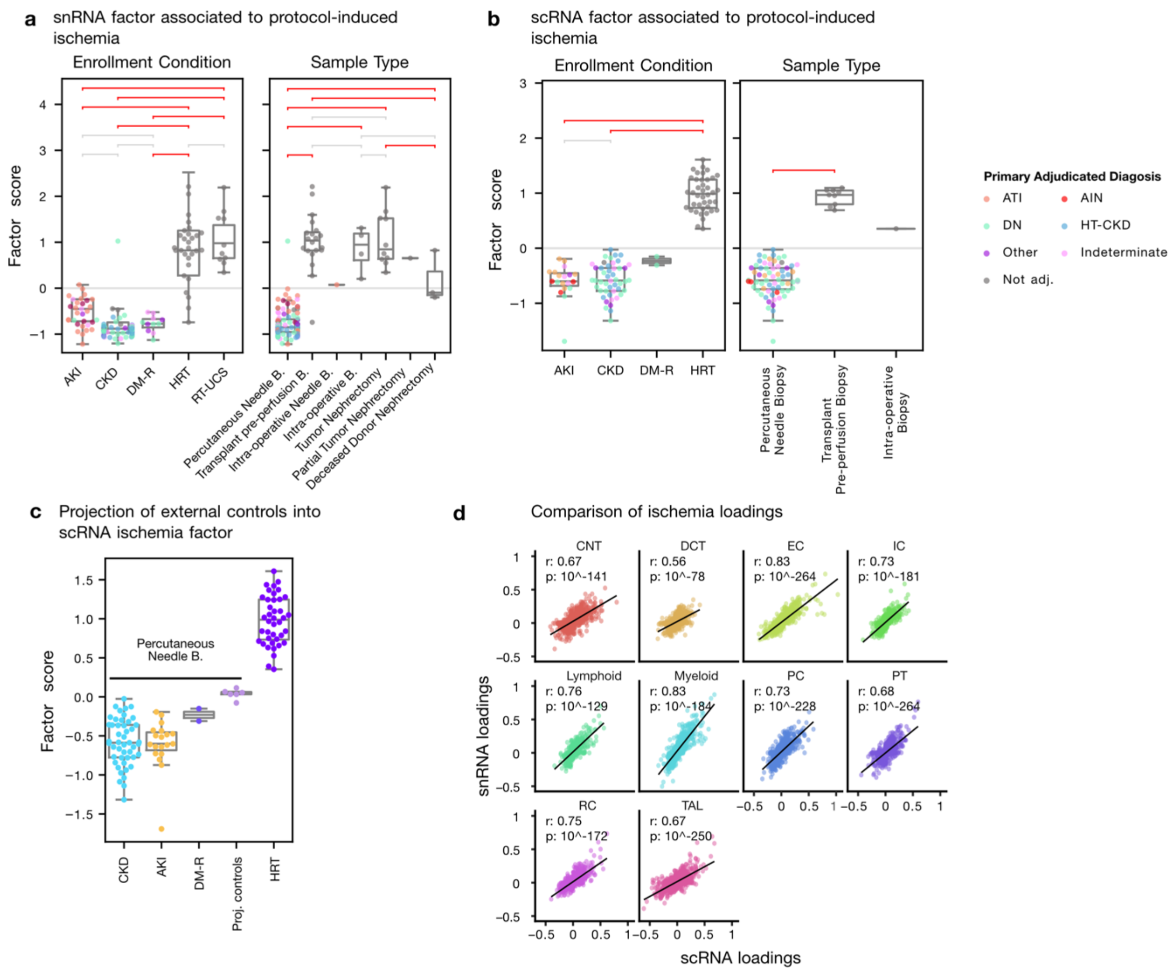

Supplementary Figure 1: Factors capture ischemic signature due to procurement

a./b. Technical factors in snRNA and scRNA separate samples based on enrollment conditions, and is confounded with sample procurement method c. Projection of healthy reference samples from the CROCODILE study (percutaneous biopsies) onto scRNA factor capturing ischemia d. Correlation between factor loadings from snRNA and scRNA for factors relating to ischemia signature

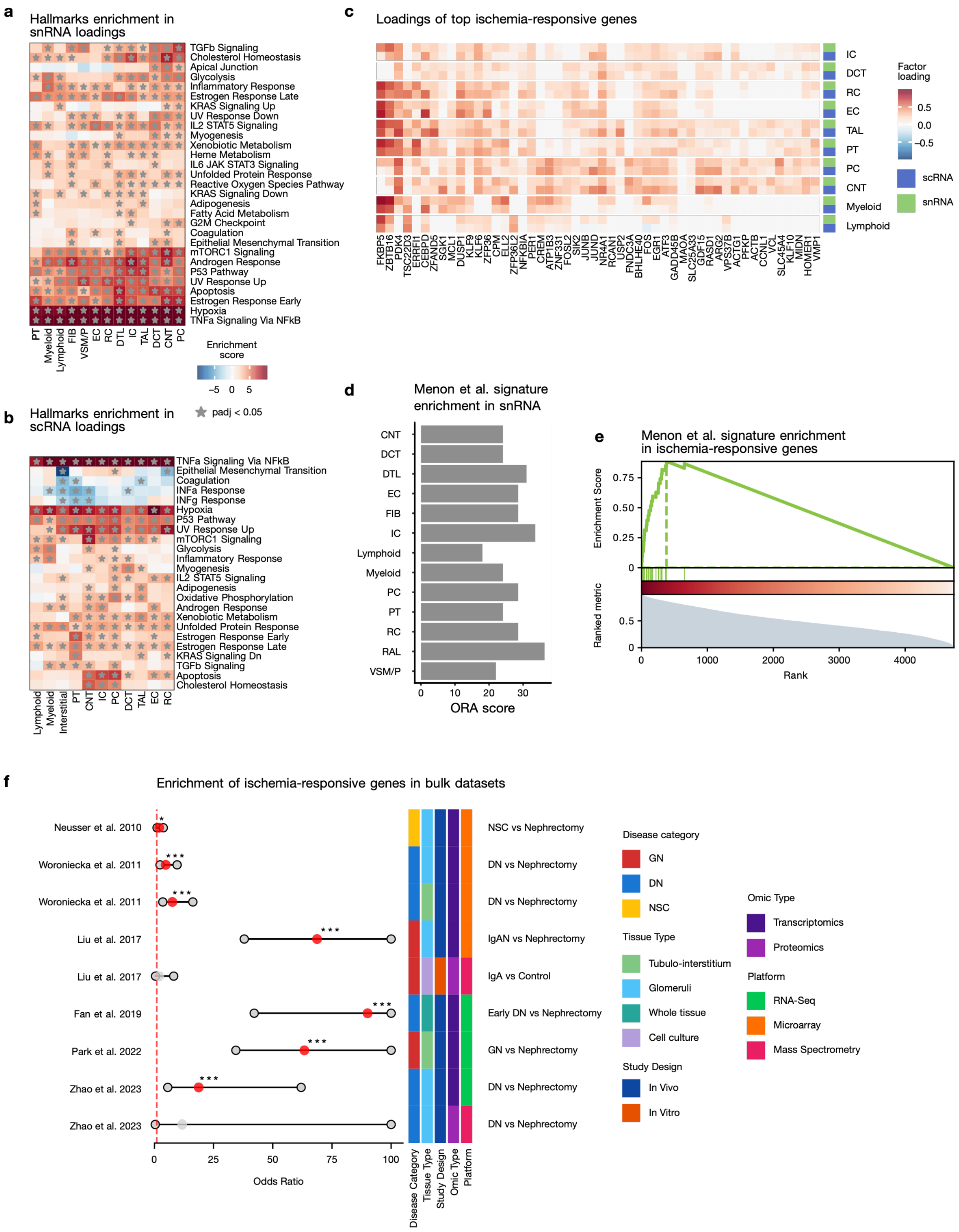

Supplementary Figure 2: Ischemic signature is captured by factors in snRNA and scRNA

a./b. MSigDB hallmark enrichment in factor shows increased inflammatory signaling, hypoxia and apoptosis in snRNA and scRNA respectively c. Ischemia-induced gene signature based on consensus between snRNA and scRNA factor

loadings across cell types d. Enrichment of gene signature from Menon et al.[28] *(derived from scRNA) in the factor loadings of snRNA e. GSEA running score in the ischemia ranking shown in c. of the signature from Menon et al. f. Odds ratio of finding ischemia signature genes (from c) in genes more highly expressed in controls from bulk RNA and protein studies which use nephrectomy samples as control.*

Supplementary Table S1: scRNA cohort characteristics

		**AKI**	**CKD**	**DM-R**
	**N**	**19**	**46**	**2**
**General demographics**	Age	55.00 [35.50; 62.00]	62.50 [54.50; 66.75]	44.50 [38.25; 50.75]
	BMI	25.61 [23.55; 29.45]	30.86 [28.27; 36.22]	35.45 [29.32; 41.57]
	Female	7 (36.8%)	23 (50.0%)	1 (50.0%)
	Male	12 (63.2%)	23 (50.0%)	1 (50.0%)
	Has Diabetes	8 (42.1%)	39 (84.8%)	2 (100.0%)
	Has Hypertension	7 (36.8%)	46 (100.0%)	1 (50.0%)
**Primary Adjudicated Category**	AIN	3 (15.8%)	0 (0.0%)	0 (0.0%)
	ATI	8 (42.1%)	0 (0.0%)	0 (0.0%)
	DN	4 (21.1%)	19 (41.3%)	2 (100.0%)
	HT-CKD	0 (0.0%)	14 (30.4%)	0 (0.0%)
	Indeterminate	3 (15.8%)	8 (17.4%)	0 (0.0%)
	Other	1 (5.3%)	4 (8.7%)	0 (0.0%)
	Not Adj.	0 (0.0%)	1 (2.2%)	0 (0.0%)
**Lab values**	Most Recent eGFR	51.43 [36.53; 79.06]	45.61 [38.18; 58.18]	114.29 [109.56; 119.02]
	Urinary ACR	2.73 [2.40; 3.02]	2.60 [1.51; 3.39]	0.95 [0.89; 1.01]
	Urinary PCR	2.74 [2.41; 3.29]	3.11 [2.31; 3.42]	N/A
	Most Recent HbA1c	6.00 [5.50; 8.05]	7.10 [6.30; 7.95]	8.25 [8.12; 8.38]
**Treatments**	On SGLT2 Inhibitor	1 (5.3%)	13 (28.3%)	0 (0.0%)
	On Insulin	2 (10.5%)	22 (47.8%)	2 (100.0%)
	On RAAS Inhibitor	3 (15.8%)	39 (84.8%)	1 (50.0%)
	On Diuretics	4 (21.1%)	22 (47.8%)	0 (0.0%)
	On ACEi/ARB	3 (15.8%)	37 (80.4%)	1 (50.0%)
	On Metformin	2 (10.5%)	15 (32.6%)	0 (0.0%)
**TIV scoring**	Interstitial Fibrosis (%)	0.30 [0.07; 0.55]	0.28 [0.10; 0.40]	0.01 [0.01; 0.01]
	Tubular Atrophy (%)	0.15 [0.04; 0.53]	0.20 [0.10; 0.31]	0.01 [0.00; 0.01]
	Tubular Injury (%)	0.20 [0.10; 0.40]	0.20 [0.10; 0.30]	0.25 [0.18; 0.33]
**All adjudication findings**	Acute Tubular Injury	15.0 (78.9%)	18.0 (39.1%)	0.0 (0.0%)
	Acute Interstitial Nephritis	5.0 (26.3%)	0.0 (0.0%)	0.0 (0.0%)
	Diabetic Kidney Disease	5.0 (26.3%)	28.0 (60.9%)	2.0 (100.0%)
	Hypertensive Kidney Disease	0.0 (0.0%)	20.0 (43.5%)	0.0 (0.0%)
	Diabetic Glomerulopathy	5 (26.3%)	28 (60.9%)	2 (100.0%)
	Global Glomerulosclerosis	4.0 (21.1%)	31.0 (67.4%)	0.0 (0.0%)
	Segmental Sclerosis	5.0 (26.3%)	14.0 (30.4%)	0.0 (0.0%)

Clinical, adjudication and histology description of samples in scRNA. Continuous variables are shown as median [IQR], categorical variables as count (percentage). ACR: log2 albumin-to-creatinine ratio, PCR: log2 protein-to-creatinine ratio, eGFR: estimated glomerular filtration rate in ml/min/1.73m2. Primary adjudicated categories are mutually exclusive, whereas individual adjudication findings are not. Adjudication and TIV scoring was performed on a separate biopsy core than the one used for snRNA or scRNA. N/A: not available

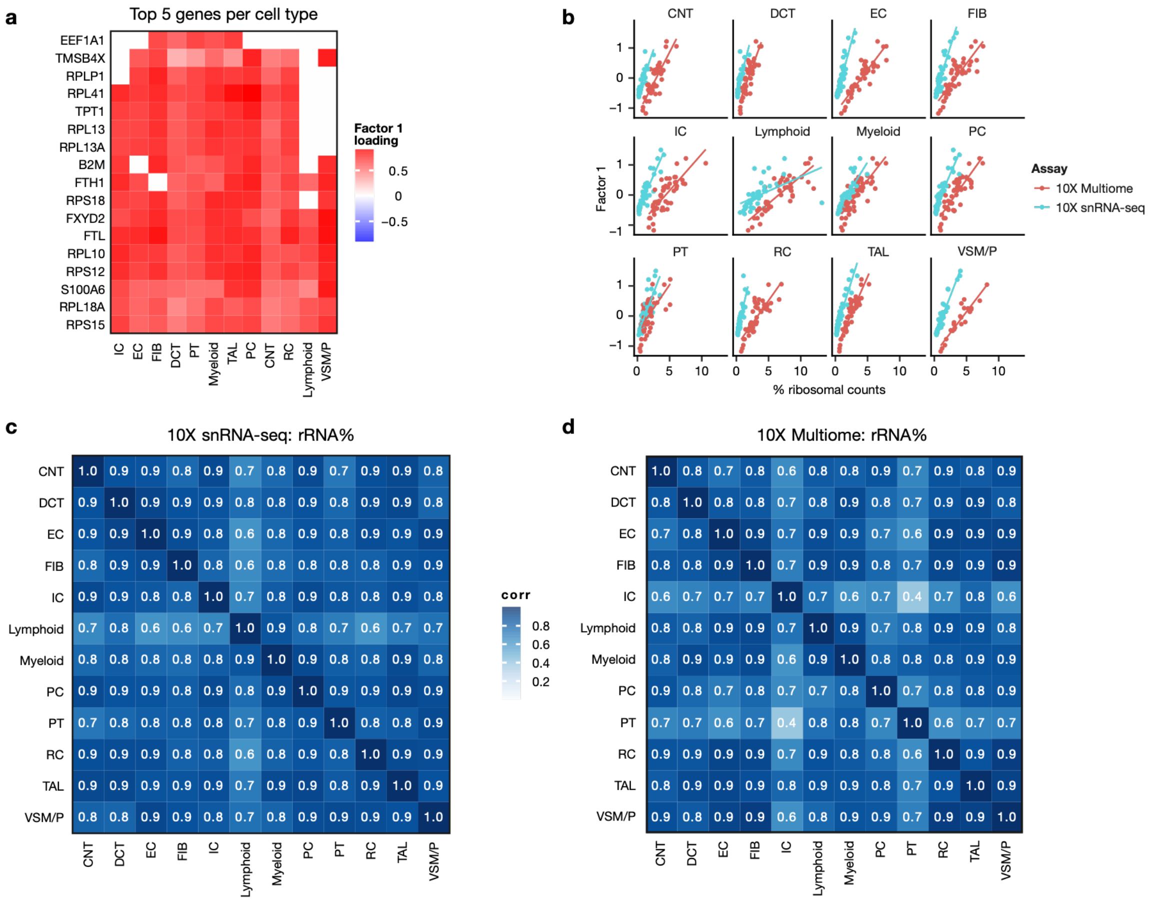

Supplementary Figure 3: Factor 1 explains ribosomal content differences between samples in snRNA

a. Top 5 Factor 1 gene loadings per cell type from snRNA. b. snRNA factor 1 scores relative to percentage of ribosomal counts c./d. Pearson correlation of ribosomal content across cell types pseudobulks in 10X snRNA-seq and 10X Multiome samples

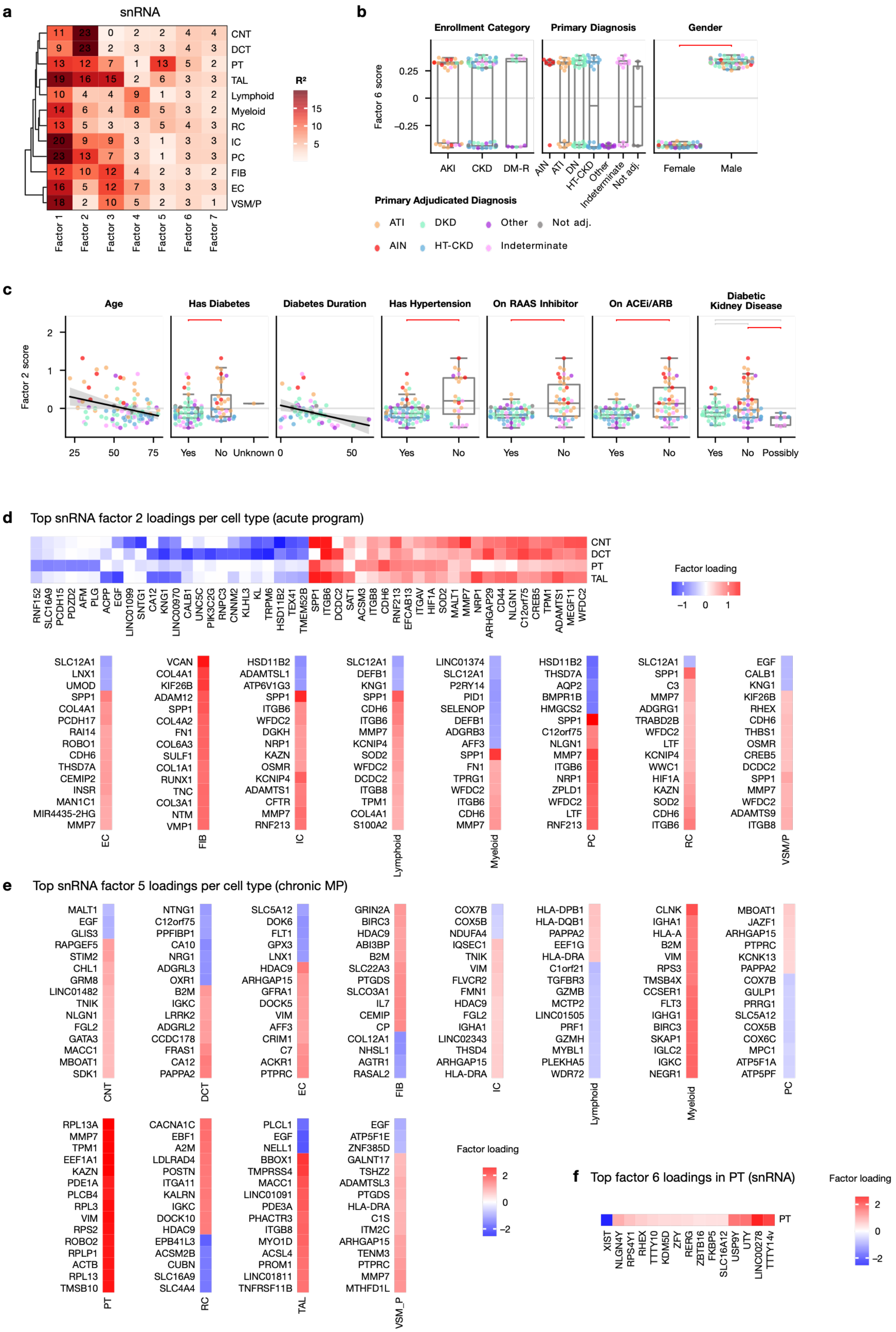
a
snRNA
CNT
DCT
PT
TAL
Lymphoid
Myeloid
RC
IC
PC
FIB
EC
VSM/P
Factor 1
Factor 2
Factor 3
Factor 4
Factor 5
Factor 6
Factor 7
R²
15
10
5
b
Enrollment Category
Primary Diagnosis
Gender
Factor 6 score
0.25
0
−0.25
AKI
CKD
DM-R
AIN
ATI
DN
HT-CKD
Other
Indeterminate
Not adj.
Female
Male
Primary Adjudicated Diagnosis
ATI
DKD
Other
Not adj.
AIN
HT-CKD
Indeterminate
c
Age
Has Diabetes
Diabetes Duration
Has Hypertension
On RAAS Inhibitor
On ACEi/ARB
Diabetic Kidney Disease
Factor 2 score
2
1
0
25
50
75
Yes
No
Unknown
0
50
Yes
No
Yes
No
Yes
No
Yes
No
Possibly
d
Top snRNA factor 2 loadings per cell type (acute program)
CNT
DCT
PT
TAL
Factor loading
−1
0
1
RNF152 SLC16A9 PCDH15 PDZD2 AFM PLG ACPP EGF LINC01099 SNTG1 CA12 KNG1 LINC00970 CALB1 UNC5C PIK3C2G RNPC3 CNNM2 KLHL3 KL TRPM6 HSD11B2 TEX41 TMEM52B SPP1 ITGB6 DCDC2 SAT1 ACSM3 ITGB8 CDH6 RNF213 EFCAB13 ITGAV HIF1A SOD2 MALT1 MMP7 NRP1 ARHGAP29 CD44 NLGN1 C12orf75 CREB5 TPM1 ADAMTS1 MEGF11 WFDC2
EC: SLC12A1 LNX1 UMOD SPP1 COL4A1 PCDH17 RAI14 ROBO1 CDH6 THSD7A CEMIP2 INSR MAN1C1 MIR4435-2HG MMP7
FIB: VCAN COL4A1 KIF26B ADAM12 SPP1 COL4A2 FN1 COL6A3 SULF1 COL1A1 RUNX1 TNC COL3A1 NTM VMP1
IC: HSD11B2 ADAMTSL1 ATP6V1G3 SPP1 ITGB6 WFDC2 DGKH NRP1 KAZN OSMR KCNIP4 ADAMTS1 CFTR MMP7 RNF213
Lymphoid: SLC12A1 DEFB1 KNG1 SPP1 CDH6 ITGB6 MMP7 KCNIP4 SOD2 WFDC2 DCDC2 ITGB8 TPM1 COL4A1 S100A2
Myeloid: LINC01374 SLC12A1 P2RY14 PID1 SELENOP DEFB1 ADGRB3 AFF3 SPP1 FN1 TPRG1 WFDC2 ITGB6 CDH6 MMP7
PC: HSD11B2 THSD7A AQP2 BMPR1B HMGCS2 SPP1 C12orf75 NLGN1 MMP7 ITGB6 NRP1 ZPLD1 WFDC2 LTF RNF213
RC: SLC12A1 SPP1 C3 MMP7 ADGRG1 TRABD2B WFDC2 LTF KCNIP4 WWC1 HIF1A KAZN SOD2 CDH6 ITGB6
VSM/P: EGF CALB1 KNG1 KIF26B RHEX CDH6 THBS1 OSMR CREB5 DCDC2 SPP1 MMP7 WFDC2 ADAMTS9 ITGB8
e
Top snRNA factor 5 loadings per cell type (chronic MP)
CNT: MALT1 EGF GLIS3 RAPGEF5 STIM2 CHL1 GRM8 LINC01482 TNIK NLGN1 FGL2 GATA3 MACC1 MBOAT1 SDK1
DCT: NTNG1 C12orf75 PPFIBP1 CA10 NRG1 ADGRL3 OXR1 B2M IGKC LRRK2 ADGRL2 CCDC178 FRAS1 CA12 PAPPA2
EC: SLC5A12 DOK6 FLT1 GPX3 LNX1 HDAC9 ARHGAP15 GFRA1 DOCK5 VIM AFF3 CRIM1 C7 ACKR1 PTPRC
FIB: GRIN2A BIRC3 HDAC9 ABI3BP B2M SLC22A3 PTGDS SLCO3A1 IL7 CEMIP CP COL12A1 NHSL1 AGTR1 RASAL2
IC: COX7B COX5B NDUFA4 IQSEC1 TNIK VIM FLVCR2 FMN1 HDAC9 FGL2 IGHA1 LINC02343 THSD4 ARHGAP15 HLA-DRA
Lymphoid: HLA-DPB1 HLA-DQB1 PAPPA2 EEF1G HLA-DRA C1orf21 TGFBR3 GZMB MCTP2 LINC01505 PRF1 GZMH MYBL1 PLEKHA5 WDR72
Myeloid: CLNK IGHA1 HLA-A B2M VIM RPS3 TMSB4X CCSER1 FLT3 IGHG1 BIRC3 SKAP1 IGLC2 IGKC NEGR1
PC: MBOAT1 JAZF1 ARHGAP15 PTPRC KCNK13 PAPPA2 COX7B GULP1 PRRG1 SLC5A12 COX5B COX6C MPC1 ATP5F1A ATP5PF
PT: RPL13A MMP7 TPM1 EEF1A1 KAZN PDE1A PLCB4 RPL3 VIM RPS2 ROBO2 RPLP1 ACTB RPL13 TMSB10
RC: CACNA1C EBF1 A2M LDLRAD4 POSTN ITGA11 KALRN IGKC DOCK10 HDAC9 EPB41L3 ACSM2B CUBN SLC16A9 SLC4A4
TAL: PLCL1 EGF NELL1 BBOX1 TMPRSS4 MACC1 LINC01091 PDE3A PHACTR3 ITGB8 MYO1D ACSL4 PROM1 LINC01811 TNFRSF11B
VSM_P: EGF ATP5F1E ZNF385D GALNT17 TSHZ2 ADAMTSL3 PTGDS HLA-DRA C1S ITM2C ARHGAP15 TENM3 PTPRC MMP7 MTHFD1L
Factor loading
2
0
−2
f
Top factor 6 loadings in PT (snRNA)
PT
XIST NLGN4Y RPS4Y1 RHEX TTTY10 KDM5D ZFY RERG ZBTB16 FKBP5 SLC16A12 USP9Y UTY LINC00278 TTTY14
Factor loading
2
0
−2

Supplementary Figure 4:

a. Gene expression variance explained by MCFA model in snRNA per cell type b. Factor 6 captures sex differences c. additional metadata associations with Factor 2 of snRNA. Horizontal brackets show Tukey's HSD tests in red if $p < 0.05$ and grey if not significant. CBD: Cannot be Determined d. Top gene loadings in factor 2 (acute program) per cell type e. Top loadings in factor 5 (chronic program) per cell type f. Top loadings in factor 6 in PT

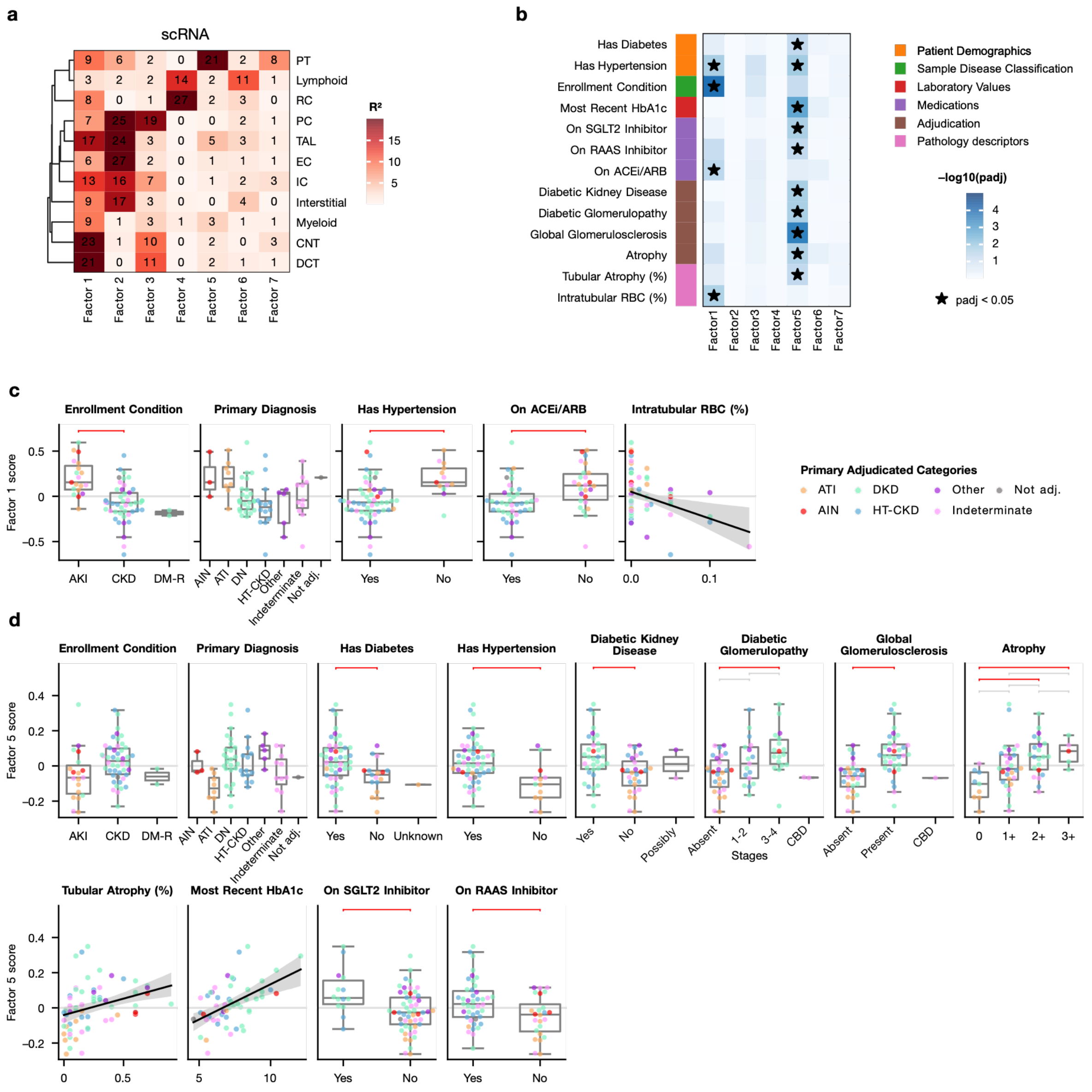

Supplementary Figure 5:

*a. Gene expression variance explained by MCFA model in scRNA per cell type b. Statistical significance of the association between sample metadata and the MCFA factor scores in scRNA; * marks significant associations (adj. p. < 0.05). c./d. Metadata associations with Factor 1 and 5 respectively (acute, chronic) of scRNA. Horizontal brackets show Tukey's HSD tests in red if p < 0.05 and grey if not significant. CBD: Cannot be Determined*

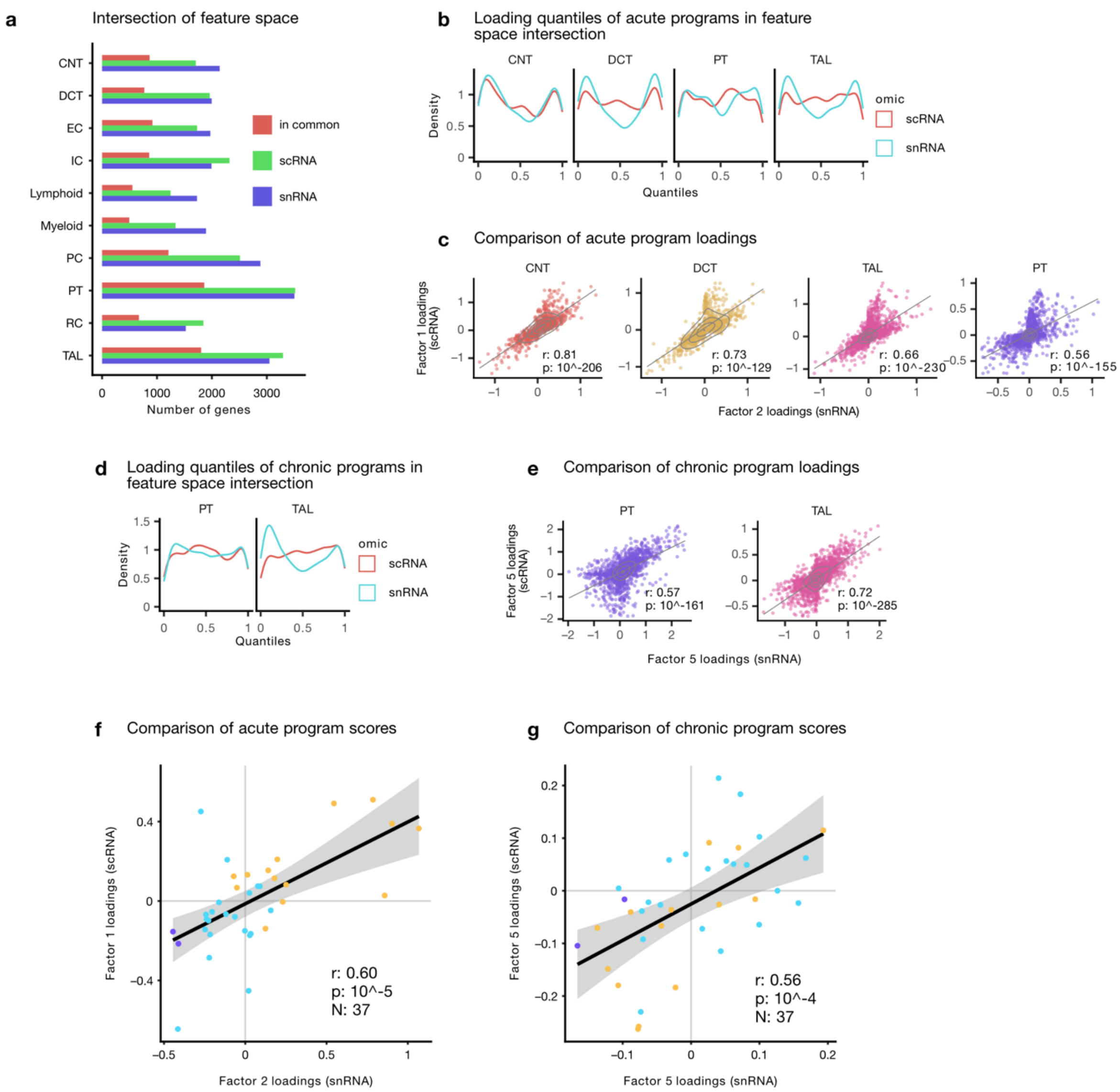

Supplementary Figure 6: Comparison of factors from snRNA and scRNA

a. Gene overlap between snRNA and scRNA MCFA models, b. quantiles of gene loadings for genes included in the intersection of genes between snRNA factor 2 and scRNA factor 1 (acute programs) for best explained cell types, c. Pearson correlation of the gene loadings between snRNA factor 2 and scRNA factor 1 (acute programs), d. as in b. for factors 5 (chronic programs) from snRNA/scRNA, e. as in c. for factors 5 (chronic programs) from snRNA/scRNA, f. Pearson correlation of snRNA factor 2 and scRNA factor 1 scores (acute programs) for patients with both snRNA and scRNA data , g. as in f. for factors 5 (chronic programs) from snRNA/scRNA

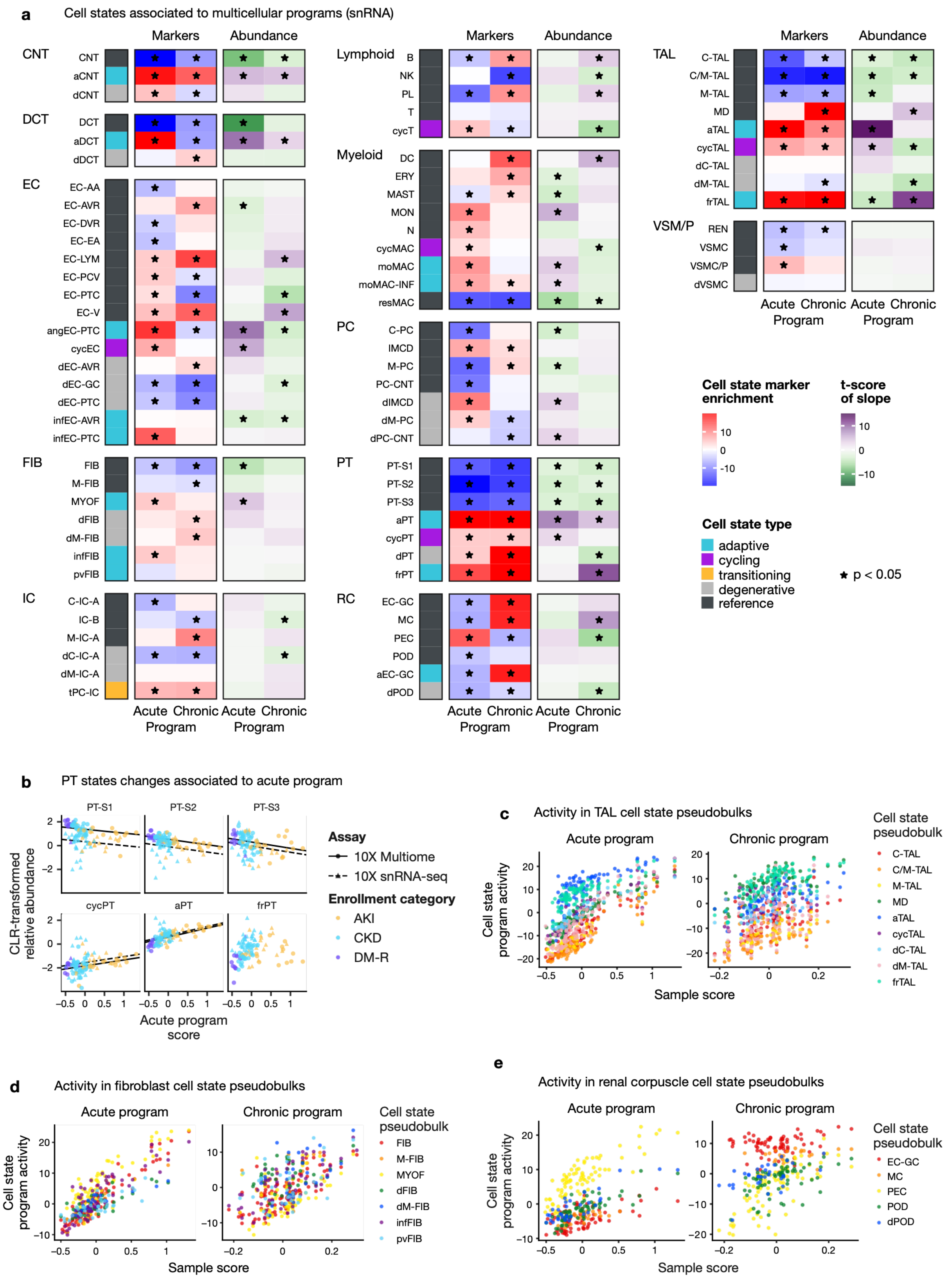

Supplementary Figure 7: Compositional and molecular shifts captured by acute and chronic

programs in snRNA

*a. Cell state marker enrichment scores (red-blue heatmaps) in program loadings and t-scores of the slopes associating (e.g., in b) within-cell type relative cell state abundance to sample program scores (purple-green heatmaps) for major cell types in snRNA. * marks enrichments or slopes with padj < 0.05 b. CLR-transformed cell state proportion within PT relative to the acute sample program score. Regression lines show slope of the associated mixed-effect model, c-e acute and chronic program activity in PT, TAL and renal corpuscle cell state-level pseudobulks by sample program score in snRNA*

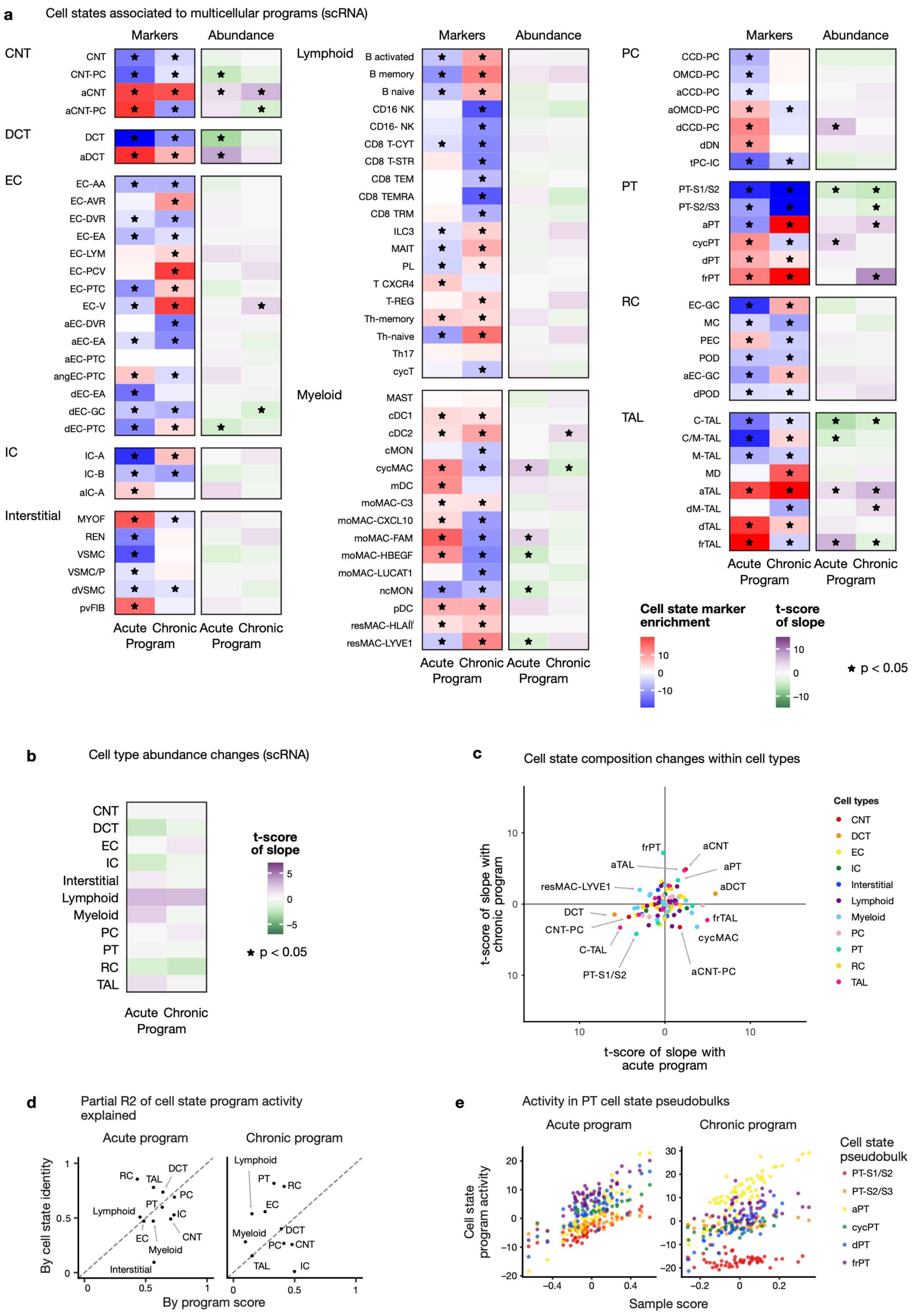
a
Cell states associated to multicellular programs (scRNA)
Markers
Abundance
CNT
CNT
CNT-PC
aCNT
aCNT-PC
DCT
DCT
aDCT
EC
EC-AA
EC-AVR
EC-DVR
EC-EA
EC-LYM
EC-PCV
EC-PTC
EC-V
aEC-DVR
aEC-EA
aEC-PTC
angEC-PTC
dEC-EA
dEC-GC
dEC-PTC
IC
IC-A
IC-B
aIC-A
Interstitial
MYOF
REN
VSMC
VSMC/P
dVSMC
pvFIB
Acute Chronic Acute Chronic
Program Program
Lymphoid
B activated
B memory
B naive
CD16 NK
CD16- NK
CD8 T-CYT
CD8 T-STR
CD8 TEM
CD8 TEMRA
CD8 TRM
ILC3
MAIT
PL
T CXCR4
T-REG
Th-memory
Th-naive
Th17
cycT
Myeloid
MAST
cDC1
cDC2
cMON
cycMAC
mDC
moMAC-C3
moMAC-CXCL10
moMAC-FAM
moMAC-HBEGF
moMAC-LUCAT1
ncMON
pDC
resMAC-HLAII
resMAC-LYVE1
PC
CCD-PC
OMCD-PC
aCCD-PC
aOMCD-PC
dCCD-PC
dDN
tPC-IC
PT
PT-S1/S2
PT-S2/S3
aPT
cycPT
dPT
frPT
RC
EC-GC
MC
PEC
POD
aEC-GC
dPOD
TAL
C-TAL
C/M-TAL
M-TAL
MD
aTAL
dM-TAL
dTAL
frTAL
Cell state marker enrichment
10
0
-10
t-score of slope
10
0
-10
★ p < 0.05
b
Cell type abundance changes (scRNA)
CNT
DCT
EC
IC
Interstitial
Lymphoid
Myeloid
PC
PT
RC
TAL
t-score of slope
5
0
−5
★ p < 0.05
Acute Chronic
Program
c
Cell state composition changes within cell types
t-score of slope with chronic program
t-score of slope with acute program
frPT
aCNT
aTAL
aPT
resMAC-LYVE1
aDCT
DCT
frTAL
CNT-PC
cycMAC
C-TAL
PT-S1/S2
aCNT-PC
Cell types
CNT
DCT
EC
IC
Interstitial
Lymphoid
Myeloid
PC
PT
RC
TAL
d
Partial R2 of cell state program activity explained
Acute program
Chronic program
By cell state identity
By program score
RC
TAL
DCT
PC
PT
Lymphoid
IC
CNT
EC
Myeloid
Interstitial
e
Activity in PT cell state pseudobulks
Acute program
Chronic program
Cell state program activity
Sample score
Cell state pseudobulk
PT-S1/S2
PT-S2/S3
aPT
cycPT
dPT
frPT

Supplementary Figure 8: Compositional and molecular shifts captured by acute and chronic programs in scRNA

*a. Cell state marker enrichment scores (red-blue heatmaps) in program loadings and t-scores of the slopes associating within-cell type relative cell state abundance to sample program scores (purple-green heatmaps) for major cell types in scRNA. * marks enrichments or slopes with padj < 0.05 b. Associations of acute and chronic program scores to overall shifts in relative cell type abundance in scRNA * mark significant associations (adj. p. < 0.05) c. t-scores of the slopes from mixed-effect model associating sample level acute (x-axis) and chronic (y-axis) program scores with relative cell state abundance within the respective cell types d. Partial R2 contribution of cell state identity and sample program score to the prediction of program activity in cell state pseudobulks in scRNA (e.g., show in e) e. Acute and chronic program activity in PT cell state-level pseudobulks by sample program score in scRNA*

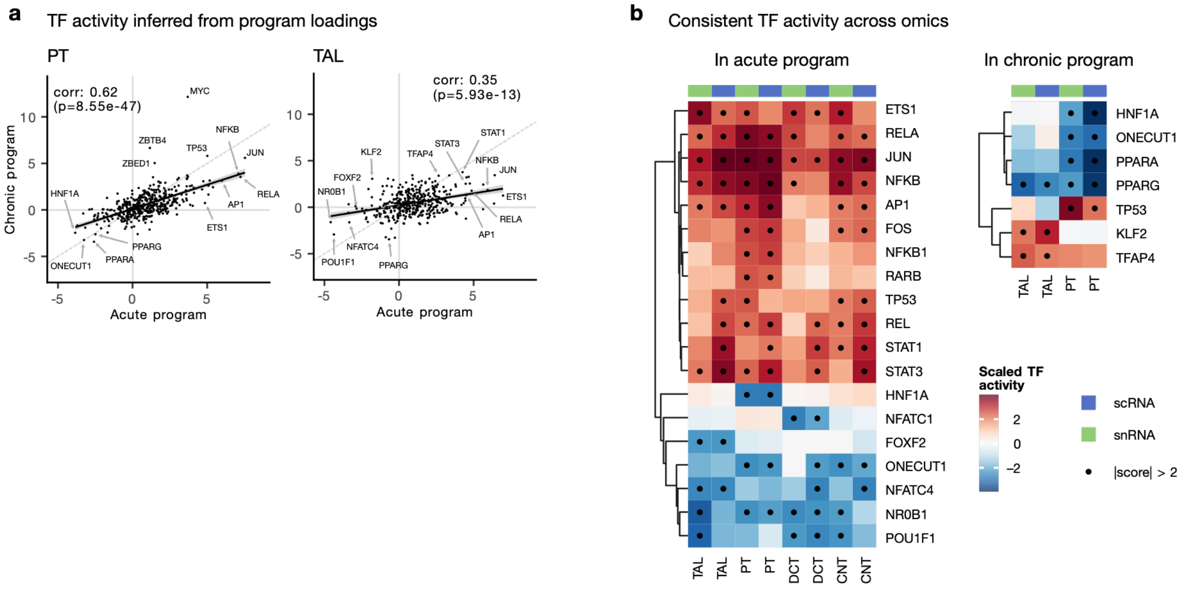

Supplementary Figure 9:

a. Correlation between transcription factor activities in the loadings of the acute (left) and chronic (right) programs for PT and TAL b. Top most deregulated transcription factors (absolute z-standardized activity > 2) in common between snRNA and scRNA for the acute (left) and chronic (right) program

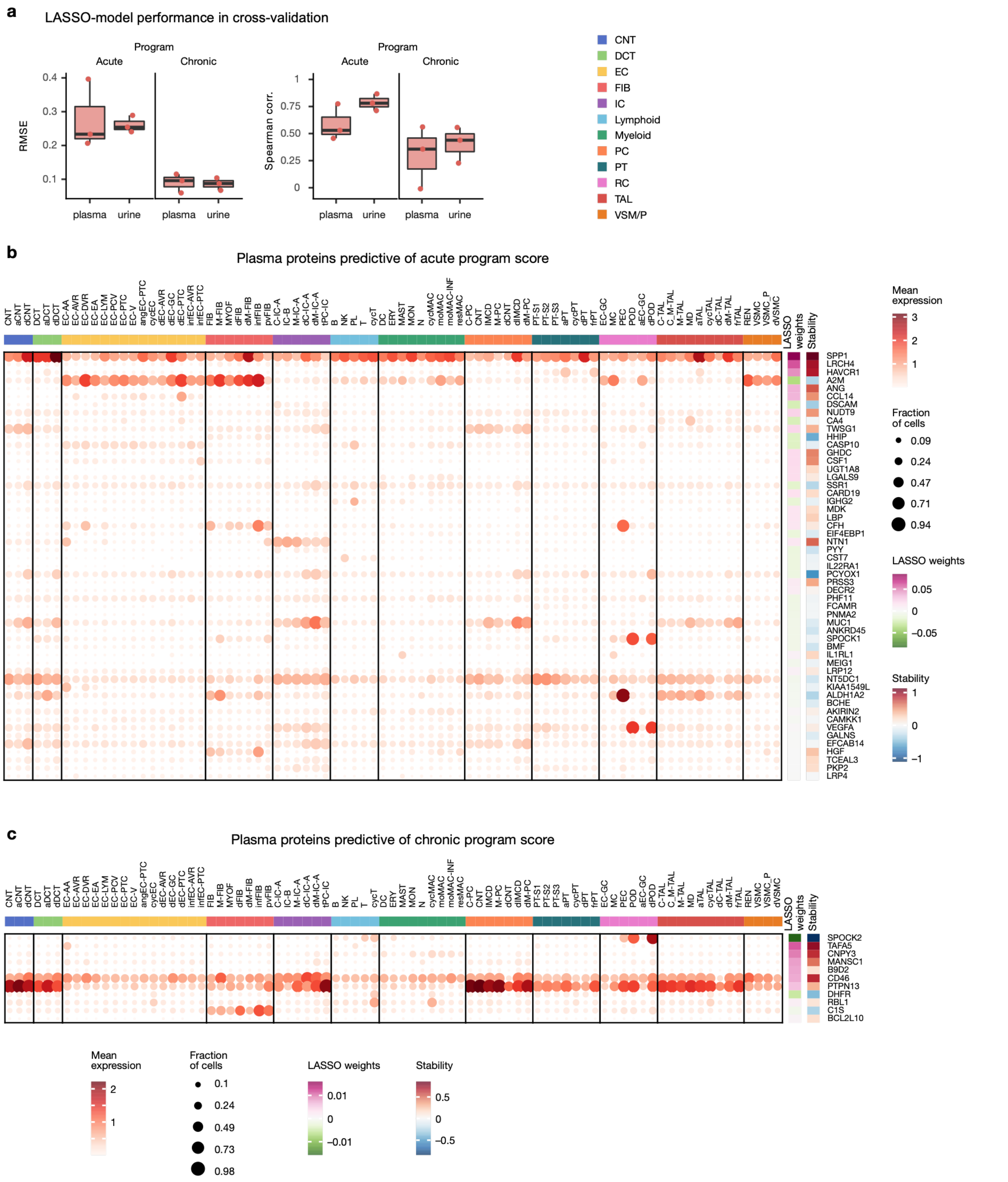

Supplementary Figure 10: Cell type and cell state specificity of expression of plasma proteins predictive of acute and chronic program scores

a. LASSO-model performance in cross-validation in terms of root mean-square error (RMSE) and spearman correlation to predict acute/chronic program scores of samples from plasma or urine somascan proteins b./c. gene

expression of plasma proteins that are cell type or cell state markers (dotplot), which were selected by the LASSO model to predict the acute and chronic program scores, respectively. The LASSO weights are in SD units for each protein. The stability is based on the mean/sd LASSO weight in bootstrap resampling times the inclusion rate (higher absolute value shows better numerical stability)

Supplementary Table S2: Somascan data characteristics and models

	**Urine**	**Plasma**
**Features**		
Total measured	7404	7596
After QC	5722	5431
Acute model	20	61
Chronic model	9	13
**Samples**		
Total measured	137	137
After QC	137	121
Acute and chronic models	69	62
Without snRNA	68	59

Total number of features in the somascan data, after filtering, and finally included in the acute and chronic LASSO models. Total number of samples with somascan data, that pass QC and which are finally used in the LASSO models (i.e. samples with both somascan and snRNA data). Samples without snRNA were used as a validation group within KPMP.

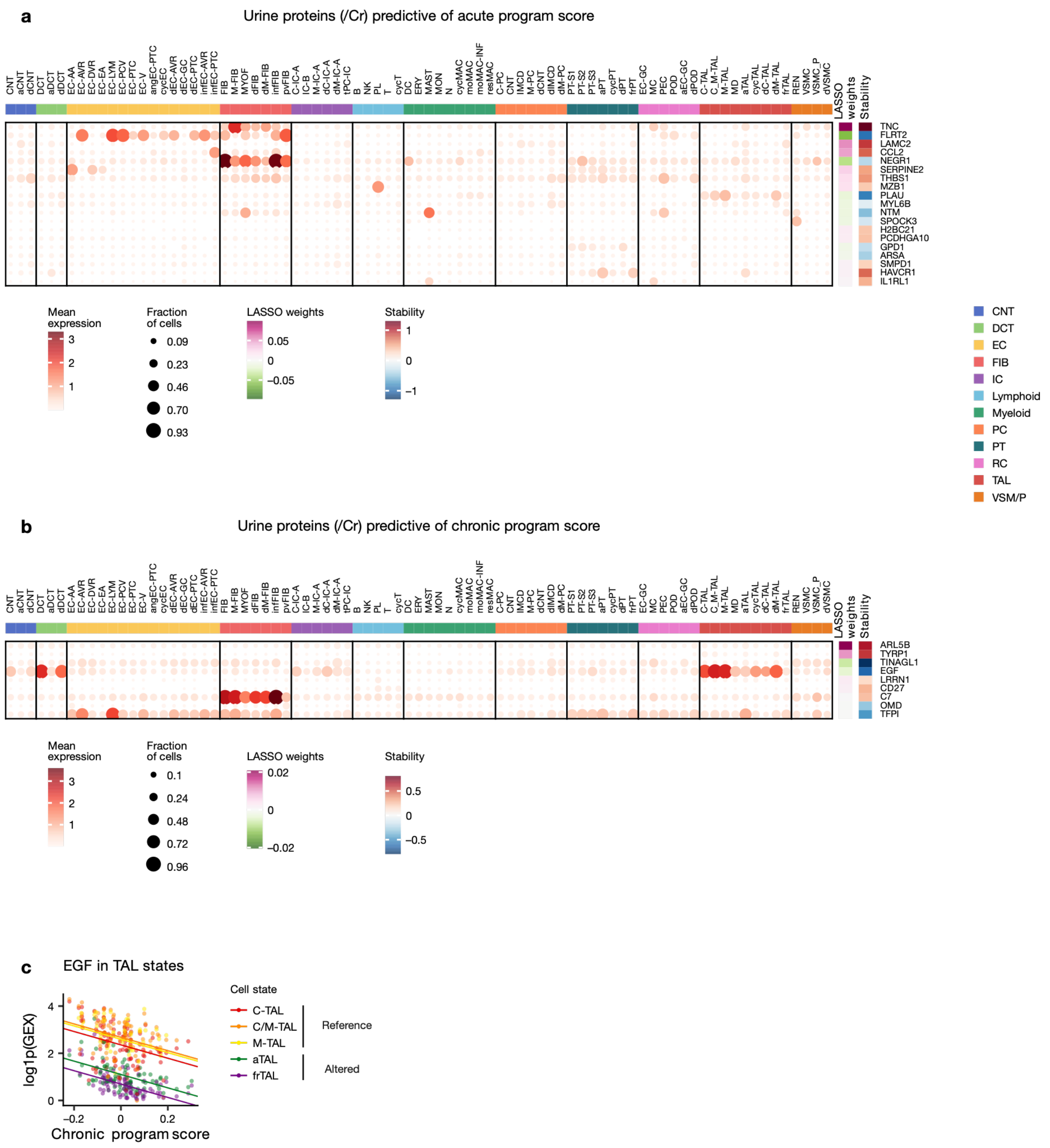

Supplementary Figure 11: Cell type and cell state specificity of expression of urine proteins predictive of acute and chronic program scores

a./b. Gene expression of urinary proteins (normalized against urinary creatinine) that are cell type or cell state markers (dotplot), which were selected by the LASSO model to predict the acute and chronic program scores, respectively. The LASSO weights are in SD units for each protein. The stability metric is the mean/sd LASSO weight in bootstrap resampling times the inclusion rate (higher absolute value shows better numerical stability) c. EGF expression in TAL cell state pseudobulks relative to chronic program score. Slopes show the relation between gene

expression and chronic program score with an intercept for each cell state, as determined by a mixed effect linear model.

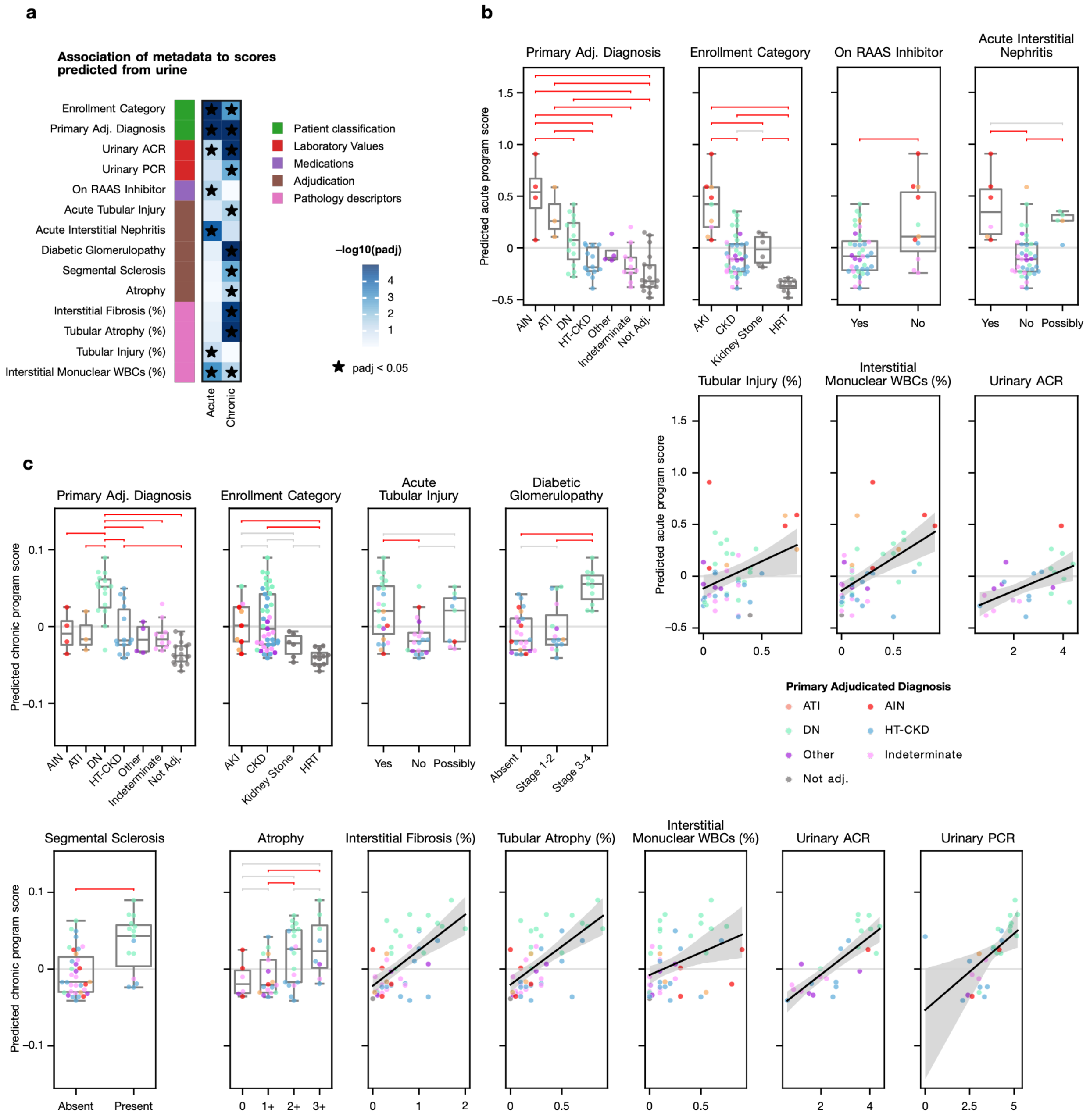

Supplementary Figure 12: Association of acute and chronic scores predicted from urinary protein abundances

*a. Significant associations between predicted scores and patient metadata. Adjusted p-values are shown based on analysis of variance or linear regression for categorical and continuous variables respectively. * marks significant*

associations (adj. p. < 0.05) b./c. Sample metadata associated with predicted acute and chronic scores. Horizontal brackets show Tukey's HSD tests in red if p < 0.05 and grey if not significant. CBD: cannot be determined.

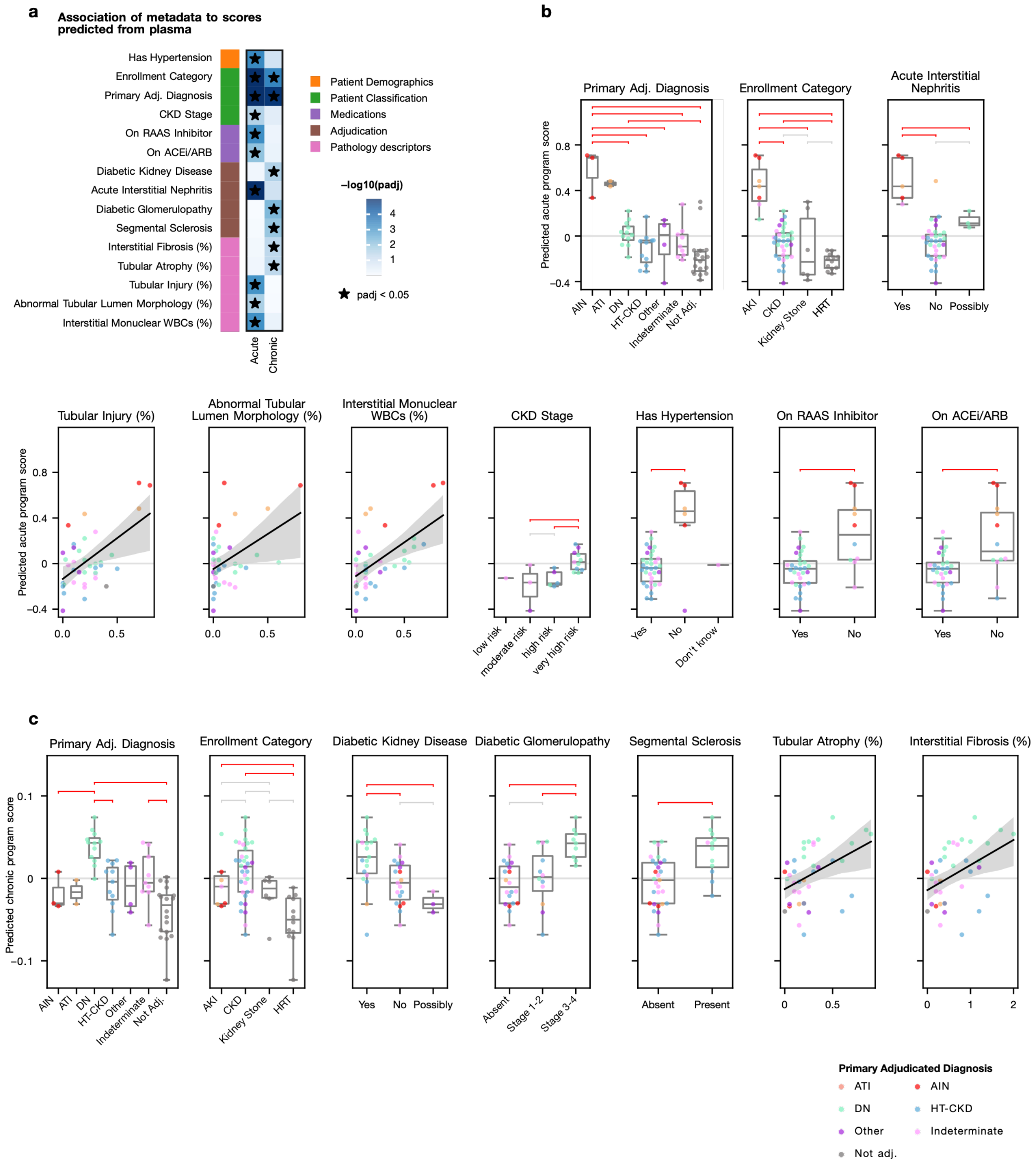

Supplementary Figure 13: Association of acute and chronic scores predicted from plasma protein abundances

*a. Significant associations between predicted scores and patient metadata. Adjusted p-values are shown based on analysis of variance or linear regression for categorical and continuous variables respectively. * marks significant*

associations (adj. p. < 0.05) b./c. Sample metadata associated with predicted acute and chronic scores. Horizontal brackets show Tukey's HSD tests in red if p < 0.05 and grey if not significant. CBD: cannot be determined.

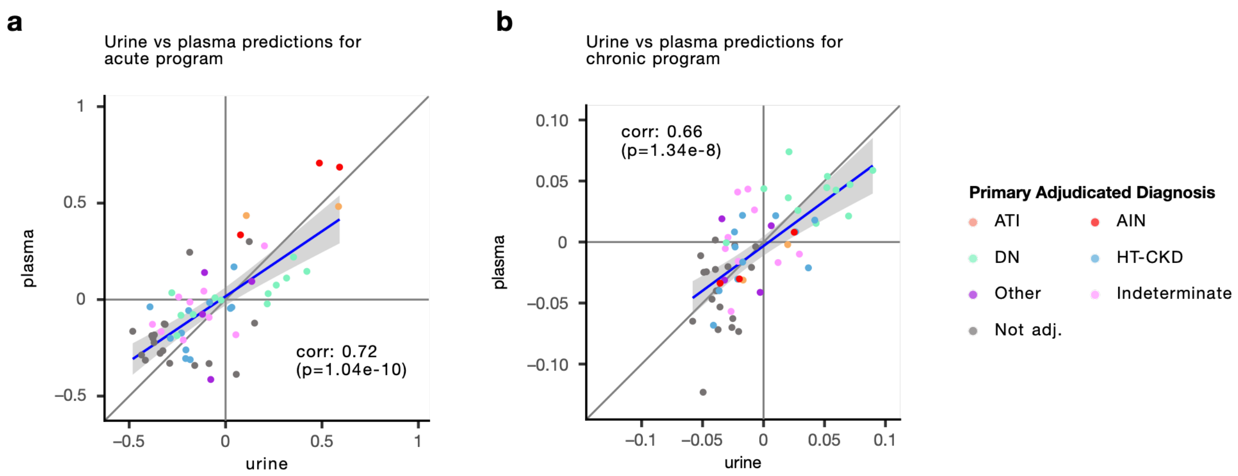

Supplementary Figure 14: Concordance between predicted acute and chronic scores

a./b. Acute and chronic scores, respectively, predicted from urine and plasma proteins

Supplementary Table S3: Baseline Characteristics of UK Biobank Participants by CKD Status.

	CKD Status at Baseline			
**Characteristic**	**Overall** N = 45167[1]	**No CKD** N = 44250[1]	**CKD** N = 917[1]	**P-value**[2]
Age (years)	56.8 (8.2)	56.7 (8.2)	62.9 (6.1)	<0.001
Sex				0.8
Female	24,416 (54%)	23,924 (54%)	492 (54%)	
Male	20,751 (46%)	20,326 (46%)	425 (46%)	
Body mass index (kg/m^2)	27.5 (4.8)	27.4 (4.8)	29.4 (5.5)	<0.001
Smoking status				<0.001
Never	24,414 (54%)	24,009 (55%)	405 (44%)	
Current/Former	20,545 (46%)	20,038 (45%)	507 (56%)	
Townsend deprivation index	-1.2 (3.2)	-1.2 (3.2)	-0.9 (3.3)	0.007
Serum creatinine (μmol/L)	72.6 (17.2)	71.4 (13.8)	127.6 (47.2)	<0.001
Serum cystatin C (mg/L)	0.9 (0.2)	0.9 (0.1)	1.5 (0.5)	<0.001
eGFR cr-based (mL/min/1.73m^2)	94.2 (13.6)	95.1 (12.1)	50.1 (9.6)	<0.001
eGFR cr-cys-based (mL/min/1.73m^2)	94.3 (15.1)	95.2 (13.8)	51.4 (13.7)	<0.001
RAS inhibitor use	5,659 (13%)	5,246 (12%)	413 (45%)	<0.001
Hypertension	12,695 (28%)	12,111 (27%)	584 (64%)	<0.001
Diabetes mellitus	2,538 (6%)	2,363 (5%)	175 (19%)	<0.001
Heart failure	475 (1%)	402 (1%)	73 (8%)	<0.001
Myocardial infarction	1,184 (3%)	1,080 (2%)	104 (11%)	<0.001
Ischemic heart disease	2,511 (6%)	2,331 (5%)	180 (20%)	<0.001

Atherosclerosis	72 (0%)	53 (0%)	19 (2%)	<0.001
Peripheral vascular disease	643 (1%)	594 (1%)	49 (5%)	<0.001
Atrial fibrillation	962 (2%)	875 (2%)	87 (9%)	<0.001
Stroke	162 (0%)	146 (0%)	16 (2%)	<0.001
COPD	1,073 (2%)	1,010 (2%)	63 (7%)	<0.001
Asthma	5,380 (12%)	5,262 (12%)	118 (13%)	0.4
Liver disease	304 (1%)	285 (1%)	19 (2%)	<0.001
Rheumatoid arthritis	662 (1%)	624 (1%)	38 (4%)	<0.001
Systemic lupus erythematosus	371 (1%)	349 (1%)	22 (2%)	<0.001

[1] Mean (SD) or n (%)

[2] Welch Two Sample t-test; Pearson's Chi-squared test

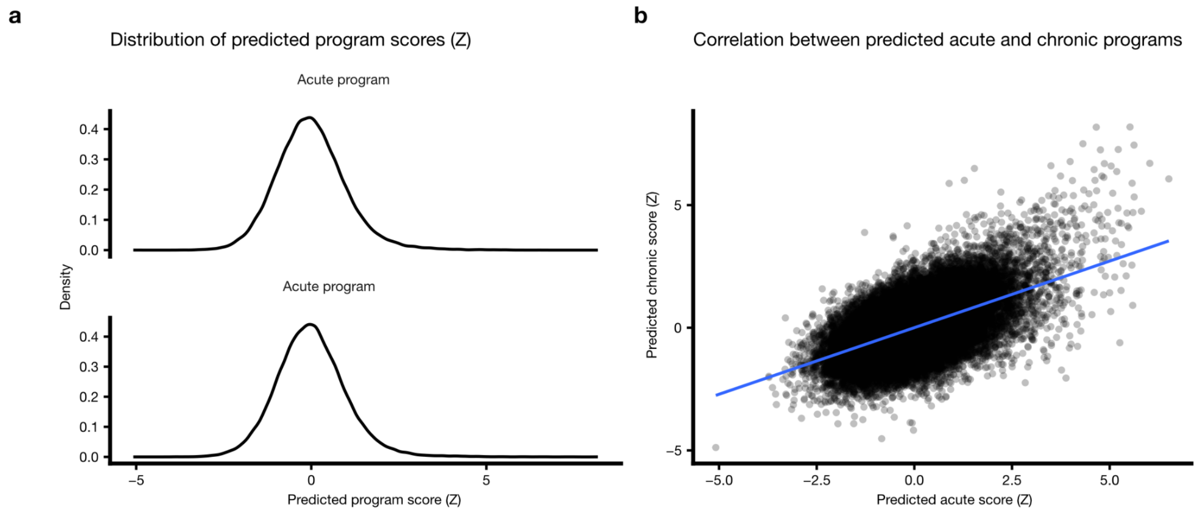

Supplementary Figure 15: Acute and chronic scores in the UK biobank

a. Distribution of acute and chronic scores (z-transformed) predicted from plasma Olink data b. Relationship between predicted acute and chronic scores in the UK biobank

Supplementary Table S4: Adjusted association between predicted program scores and eGFR.

Program	Model	N	β (95% CI)	SE	P-value
Acute	Minimal adjustment	45167	-3.10 (-3.22 to -2.99)	0.06	<0.001
Acute	Full adjustment	44749	-2.92 (-3.04 to -2.80)	0.06	<0.001
Acute	No CKD stratum	43843	-1.64 (-1.76 to -1.53)	0.06	<0.001
Acute	CKD stratum	906	-3.35 (-3.73 to -2.97)	0.19	<0.001
Acute	Main effect	44749	-1.68 (-1.79 to -1.56)	0.06	<0.001
Acute	Interaction term	44749	-1.61 (-2.09 to -1.12)	0.25	<0.001
Chronic	Minimal adjustment	45167	-5.02 (-5.12 to -4.91)	0.05	<0.001
Chronic	Full adjustment	44749	-4.88 (-4.98 to -4.77)	0.05	<0.001
Chronic	No CKD stratum	43843	-3.58 (-3.68 to -3.47)	0.05	<0.001
Chronic	CKD stratum	906	-3.60 (-3.92 to -3.27)	0.17	<0.001
Chronic	Main effect	44749	-3.59 (-3.70 to -3.49)	0.05	<0.001
Chronic	Interaction term	44749	-0.16 (-0.58 to 0.26)	0.22	0.46

Supplementary Table S5: Hazard ratio of incident AKI based on acute or chronic plasma signature in UK biobank

**Incident AKI (n = 2391/44250)**						
Time Interval	Acute HR	Acute 95% CI (Lower)	Acute 95% CI (Upper)	Chronic HR	Chronic 95% CI (Lower)	Chronic 95% CI (Upper)
0–3 months	2.14	1.31	3.49	1.49	0.82	2.72
3–6 months	2.13	1.42	3.2	2.15	1.39	3.31
6–12 months	1.82	1.23	2.68	1.59	1.04	2.44
>12 months	1.54	1.48	1.61	1.35	1.29	1.41

Supplementary Table S6: Hazard ratio of incident CKD based on acute or chronic plasma signature in UK biobank

Incident CKD (n = 2315/44250)						
Time Interval	Acute HR	Acute 95% CI (Lower)	Acute 95% CI (Upper)	Chronic HR	Chronic 95% CI (Lower)	Chronic 95% CI (Upper)
0–1 year	1.5	1.29	1.75	1.45	1.24	1.7
1–3 years	1.65	1.49	1.82	1.48	1.33	1.65
3–5 years	1.59	1.44	1.77	1.52	1.36	1.69
>5 years	1.43	1.36	1.5	1.32	1.26	1.4

Supplementary Table S7: Hazard ratio of incident AKI or CKD based on combination of acute and chronic plasma signature in UK biobank

**Phenotype Group**	**Incident AKI HR (95% CI)**	**Incident CKD HR (95% CI)**
Reference (low acute, low chronic)	1.00 (reference)	1.00 (reference)
High Acute Only	1.48 (1.28–1.71)	1.41 (1.19–1.66)
High Chronic Only	1.07 (0.91–1.26)	1.31 (1.10–1.55)
Double Hit (high acute, high chronic)	2.13 (1.89–2.41)	2.23 (1.94–2.56)
